# 贝舒地尔治疗重度慢性移植物抗宿主病20例的疗效与安全性

**DOI:** 10.3760/cma.j.cn121090-20241017-00401

**Published:** 2025-08

**Authors:** 智 王, 建华 游, 文婷 陈, 婷婷 邢, 依 罗, 晓冬 莫, 炯 胡

**Affiliations:** 1 上海交通大学医学院附属瑞金医院海南医院，琼海 571400 Hainan Hospital of Ruijin Hospital Affiliated to Shanghai Jiao Tong University School of Medicine, Qionghai 571400, China; 2 上海交通大学医学院附属瑞金医院，上海 200025 Ruijin Hospital Affiliated to Shanghai Jiao Tong University School of Medicine, Shanghai 200025, China; 3 海南省人民医院，海口 570311 Hainan General Hospital, Haikou 570311, China; 4 海南大学生物医学工程学院，海口 570228 Hainan University School of Biomedical Engineering, Haikou 570228, China; 5 浙江大学医学院附属第一医院，杭州 311100 The First Affiliated Hospital, Zhejiang University School of Medicine, Hangzhou 311100, China; 6 北京大学人民医院，北京大学血液病研究所，国家血液系统疾病临床医学研究中心，造血干细胞移植治疗血液病北京市重点实验室，北京 100044 Peking University People's Hospital, Peking University Institute of Hematology, National Clinical Research Center for Hematologic Disease, Beijing Key Laboratory of Hematopoietic Stem Cell Transplantation, Beijing 100044, China

**Keywords:** 贝舒地尔, ROCK2抑制剂, 慢性移植物抗宿主病, 异基因造血干细胞移植, Belumosudil, ROCK2 Inhibitor, Chronic graft-versus-host disease, Allo-HSCT

## Abstract

**目的:**

评估贝舒地尔治疗慢性移植物抗宿主病（cGVHD）的疗效和安全性。

**方法:**

收集2023年5月至2024年3月期间在上海交通大学医学院附属瑞金医院海南医院接受贝舒地尔治疗的cGVHD病例，回顾性分析治疗的总缓解率、各器官缓解率、起效时间、Lee症状量表（LSS）得分变化、糖皮质激素的减量或停用、无失败生存（FFS）以及不良事件发生情况。

**结果:**

共纳入20例接受贝舒地尔治疗的cGVHD患者，男15例，女5例，中位年龄34.5（12～67）岁，其中3例患者≤18岁。所有患者均为重度cGVHD，18例（90.0％）患者≥4个器官受累。既往中位治疗线数为4线，其中15例（75％）既往接受过芦可替尼治疗。所有患者接受贝舒地尔200 mg每日1次联合其他cGVHD系统性治疗。总缓解率为90.0％（95％ *CI*：68.3％～98.8％），均为部分缓解，所有受累器官均有患者实现完全缓解。中位起效时间为1.6（0.9～8.4）个月。中位随访5.0（1.4～9.8）个月。16例（80.0％）患者在3个月时LSS评分达到临床意义的改善，42.6％（6/14）的患者减少了糖皮质激素用量，无失败生存率为79.6％（95％ *CI*：61.4％～100.0％）。治疗期间2例患者发生≥3级不良事件。

**结论:**

贝舒地尔对重度cGVHD患者具有良好的疗效和安全性。

## Abstract (en)

**Objective:**

To evaluate the effectiveness and safety of belumosudil for the treatment of chronic graft-versus-host disease（cGVHD）.

**Methods:**

We retrospectively collected data on patients with cGVHD who received belumosudil at Ruijin Hospital Affiliated to Shanghai Jiaotong University School of Medicine from May 2023 to March 2024. The study endpoints were overall response rate（ORR）, organ-specific response rates, time to response（TTR）, changes in Lee Symptom Scale（LSS）scores, tapering or discontinuation of corticosteroid treatment, failure-free survival（FFS）, and adverse events.

**Results:**

The study included 20 patients with cGVHD who received belumosudil, of whom 15 were men and 5 women. The median age was 34.5（12–67）years, and three patients were under 18 years old. The median follow-up duration was 5.0（1.4–9.8）months. All patients had severe cGVHD, and 18（90.0％）showed involvement of at least four organs. The median number of prior treatment lines was 4, and 15 patients（75％）had previously received ruxolitinib. All patients received 200 mg of belumosudil once daily in combination with other cGVHD systemic therapies. The ORR was 90.0％（95％ *CI* : 68.3％–98.8％）, and all responses were partial responses. The median TTR was 1.6（0.9–8.4）months. The LSS scores improved in a clinically meaningful way in 80.0％（16/20）of the patients within 3 months. The corticosteroid dose was reduced in 42.6％（6/14）of the patients. The 3-month FFS was 79.6％（95％ *CI* : 61.4％–100.0％）. Most adverse events were grade 1 or grade 2, and two patients（10.0％）experienced grade 3 or higher-grade adverse events.

**Conclusion:**

In the real-world setting, belumosudil demonstrated good effectiveness and safety in patients with cGVHD with a history of severe disease and multiorgan involvement.

慢性移植物抗宿主病（cGVHD）是异基因造血干细胞移植（allo-HSCT）后的常见并发症，其主要特征是在免疫系统重建过程中，受者组织脏器受到供者免疫系统攻击[1]。allo-HSCT是高危血液肿瘤、代谢和免疫缺陷等疾病潜在治愈手段。虽然在供者选择、预处理方案和支持治疗方面取得了重大进展，但cGVHD依然是移植后非复发死亡的主要因素之一[2]。allo-HSCT受者中，cGVHD的发生率为30％～70％[3]，中国患者中报道比例约为53％[4]。cGVHD的病理生理学过程主要为急性炎症、免疫细胞数量及功能紊乱和组织纤维化，其中纤维化是cGVHD发展的终末结局，导致器官功能障碍甚至危及生命[5]。cGVHD可累及多个器官，治疗往往需要长期使用免疫抑制剂[6]，由此带来短期和长期治疗相关毒性，严重影响生活质量，同时增加移植后晚期死亡的风险[7],[8]。目前，cGVHD一线治疗方案仍为糖皮质激素联合或不联合钙调磷酸酶抑制剂，有效率约为50％[1]，一些靶向药物如伊布替尼、芦可替尼提高了临床治疗的有效率，但感染、血小板减少和贫血等治疗相关不良反应具有较高的发生率[9]–[10]。间充质干细胞、利妥昔单抗、吗替麦考酚酯等也有一定效果，但均未取得明显优于一线方案的疗效，临床上对cGVHD尚无标准的二线治疗方案[1]。

甲磺酸贝舒地尔是一种选择性Rho卷曲螺旋蛋白激酶2（ROCK2）抑制剂，对调节炎症和纤维化反应的信号通路具有双重作用[11]–[12]。通过下调信号转导和转录激活因子（STAT）3来减少Th17细胞和滤泡性T辅助细胞，同时通过上调STAT5来增强调节性T细胞，从而在免疫反应中实现平衡。ROCK2信号通路在纤维化途径中也有重要作用，可以激活促纤维化基因的表达，从而增加胶原蛋白的产生。贝舒地尔通过阻断成纤维细胞中的ROCK2发挥调节纤维化的作用。自2021年在美国获批用于治疗cGVHD以来[13]，贝舒地尔已在中国、加拿大、澳大利亚、英国等多个国家/地区获批上市，并被欧洲血液与骨髓移植学会推荐用于治疗糖皮质无效的难治性cGVHD[14]。多项国内外研究证实贝舒地尔可作为cGVHD患者的一种新的治疗选择[15]–[17]。本研究评估贝舒地尔对既往接受过多线治疗且多个器官受累的重度cGVHD患者的疗效和安全性。

## 病例与方法

一、研究设计

本研究为回顾性研究，评价贝舒地尔在多线系统性治疗失败的重度cGVHD患者中的疗效和安全性，纳入于2023年5月至2024年3月期间，在上海交通大学医学院附属瑞金医院海南医院接受贝舒地尔治疗的20例cGVHD患者。研究数据来源于医院病历及随访，包括患者的基线特征、治疗方案以及治疗应答等信息。数据收集截止日期为2024年3月20日。

二、疗效评价及安全性分析

疗效指标包括总缓解率（ORR）、起效时间、分器官缓解率、Lee症状量表（LSS）得分变化、糖皮质激素治疗的减量或停用、以及无失败生存（FFS）。缓解定义为完全缓解（CR）或部分缓解（PR）。CR定义为每个器官的所有表现都已完全消失；PR定义为至少一个器官有改善，并且其他任何器官都没有恶化。基于2014年美国国立卫生研究院（NIH）的应答评估标准[18]，由临床医生根据患者随访频率，每1至2个月定期进行评估。LSS评分有临床意义的改善定义为较基线至少降低7分。FFS定义为在研究期间未出现新的cGVHD系统治疗、肿瘤复发或非复发性死亡。根据CTCAE 5.0版本进行不良事件的评估。

三、统计学处理

使用R4.1.2进行统计分析。使用二项分布确切概率法估计总体及分器官缓解率及相应的95％置信区间（*CI*）。使用Kaplan-Meier（K-M）方法估计FFS。对其他终点进行描述性分析。

## 结果

一、临床特征

本研究纳入20例cGVHD患者，中位年龄为34.5（12～67）岁，包括3例12～18岁的青少年。中位随访时间为5.0（1.4～9.8）个月。临床特征详见表1。启动贝舒地尔治疗时，中位cGVHD持续时间为9.0（4.0～62.0）个月。所有患者均表现为重度cGVHD。中位受累器官数为7（2～8）个，其中18例（90.0％）有≥4个器官受累。在启动贝舒地尔治疗前，10例（50.0％）接受过至少4线治疗，15例（75.0％）曾接受过芦可替尼治疗。18例（90.0％）在上线治疗后未获得缓解。

**表1:** 20例接受贝舒地尔治疗cGVHD患者的临床特征

临床特征	结果
年龄［岁，*M*（范围）］	34.5（12～67）
性别［例（％）］	
男	15（75.0）
女	5（25.0）
体重［kg，*x*±*s*］	49.4 ± 15.4
体质指数［kg/m^2^，*x*±*s*］	17.7 ± 4.1
原发疾病［例（％）］	
急性髓系白血病	8（40.0）
再生障碍性贫血	2（10.0）
急性淋巴细胞白血病	2（10.0）
慢性髓性白血病	1（5.0）
T淋巴母细胞淋巴瘤	1（5.0）
骨髓增生异常综合征	1（5.0）
其他	5（25.0）
预处理［例（％）］	
清髓性预处理	15（75.0）
未知	5（25.0）
供者来源［例（％）］	
同胞全相合	11（55.0）
单倍体	6（30.0）
无关供者	2（10.0）
其他	1（5.0）
aGVHD病史［例（％）］	
有	9（45.0）
无	11（55.0）
启动贝舒地尔治疗时器官受累数量［个，*M*（范围）］	7（2～8）
启动贝舒地尔治疗时受累器官［例（％）］	
皮肤	17（85.0）
口腔	19（95.0）
眼睛	20（100.0）
食管	17（85.0）
胃肠道	18（90.0）
肝脏	2（10.0）
肺	19（95.0）
关节筋膜	16（80.0）
既往治疗线数［例（％）］	
2	3（15.0）
3	6（30.0）
4	5（25.0）
5	0（0）
6	2（10.0）
>6	3（15.0）
未知	1（5.0）
上线治疗最佳缓疗效［例（％）］	
部分缓解	2（10.0）
无缓解	18（90.0）
既往系统性cGVHD治疗［例（％）］	
糖皮质激素	18（90.0）
钙调磷酸酶抑制剂	18（90.0）
芦可替尼	15（75.0）
吗替麦考酚酯	7（35.0）
甲氨蝶呤	5（25.0）
西罗莫司	5（25.0）
间充质干细胞	4（20.0）
巴利昔单抗	4（20.0）
伊马替尼	2（10.0）
英夫利昔单抗	2（10.0）
伊布替尼	1（5.0）
联合系统性cGVHD治疗［例（％）］	
芦可替尼	15（75.0）
糖皮质激素	14（70.0）
钙调磷酸酶抑制剂	14（70.0）
吗替麦考酚酯	2（10.0）

**注** cGVHD：慢性移植物抗宿主病；aGVHD：急性移植物抗宿主病

全部20例患者均接受200 mg每日1次贝舒地尔治疗。启动贝舒地尔治疗时联合的其他系统性cGVHD治疗包括：15例（75.0％）联合芦可替尼，14例（70.0％）联合糖皮质激素，14例（70.0％）联合钙调磷酸酶抑制剂，2例（10.0％）联合吗替麦考酚酯。其中1例在治疗2个月时死亡，1例在治疗2个月时因原发肿瘤复发而终止用药，另有1例因为断药而中断贝舒地尔治疗，其余17例患者（85.0％）持续接受贝舒地尔治疗。

二、疗效分析

在20例接受贝舒地尔治疗的患者中，18例获得缓解，ORR为90.0％（95％*CI*：68.3％～98.8％），均为部分缓解。中位起效时间为1.6（0.9～8.4）个月。受累器官的缓解情况（表2）：皮肤70.6％，口腔36.8％，眼睛75.0％，食管47.1％，胃肠道66.7％，肝脏100.0％，肺47.4％，关节筋膜50.0％。基于13例在3个月时有足够随访的患者，ORR为92.3％（95％*CI*：64.0％～99.8％）；基于8例在6个月时有足够随访的患者，ORR为100％（95％*CI*：63.1％～100％）。18例达到缓解的患者中，仅有1例在首次缓解的2个月后出现cGVHD疾病进展。基线时，14例患者联合使用糖皮质激素，等效泼尼松剂量为0.36（0.09～1.23）mg·kg^−1^·d^−1^。其中6例（42.9％）在接受贝舒地尔治疗后减量，中位降幅56.25％（33.33％～87.50％），从启动贝舒地尔治疗到糖皮质激素减量的中位间隔为30（10～60）d。16例（80.0％）在3个月时表现出LSS评分有临床意义的改善（相比基线降低≥7分），平均降低12.35分（标准差为7.67）。在18例应答者和2例非应答者中，这一比例分别为77.8％和100％。基于K-M法估计，3个月的FFS率为79.6％（95％*CI*：61.4％～100.0％）。在研究期间，共有3例患者发生治疗失败：1例患者需要新的cGVHD系统治疗，1例患者肿瘤复发，1例患者因非复发性原因死亡，均发生在启动贝舒地尔治疗3个月内。

**表2:** 20例重度慢性移植物抗宿主病患者贝舒地尔治疗后器官缓解情况

器官	总体（20例）			既往芦可替尼治疗（15例）		
	受累例数	缓解例数	缓解率［％（95％*CI*）］	受累例数	缓解例数	缓解率［％（95％*CI*）］
皮肤	17	12	70.6（44.0～89.7）	12	8	66.7（34.9～90.1）
口腔	19	7	36.8（16.3～61.6）	14	4	28.6（8.4～58.1）
眼睛	20	15	75.0（50.9～91.3）	15	11	73.3（44.9～92.2）
食管	17	8	47.1（23.0～72.2）	12	6	50.0（21.1～78.9）
胃肠道	18	12	66.7（41.0～86.7）	13	9	69.2（38.6～90.9）
肝脏	2	2	100.0（15.8～100.0）	1	1	100.0（2.5～100.0）
肺	19	9	47.4（24.4～71.1）	14	6	42.9（17.7～71.1）
关节筋膜	16	8	50.0（24.7～75.3）	11	4	36.4（10.9～69.2）

在15例芦可替尼经治患者中，13例达到缓解，ORR为86.7％（95％*CI*：59.5％～98.3％），均为部分缓解，中位起效时间为1.9（0.9～8.4）个月。基于12例在3个月时有足够随访的患者，ORR为91.7％（95％*CI*：61.5％～99.8％）；基于7例在6个月时有足够随访的患者，ORR为100％（95％*CI*：59％～100％）。11例（73.3％）在3个月时表现出了LSS评分具有临床意义的改善。贝舒地尔治疗3、6个月时总体和受累器官缓解情况见表3、表4。

**表3:** 贝舒地尔治疗3个月时总体和既往芦可替尼治疗患者受累器官缓解情况

器官	总体（13例）			既往芦可替尼治疗（12例）		
	受累例数	缓解例数	缓解率［％（95％*CI*）］	受累例数	缓解例数	缓解率［％（95％*CI*）］
皮肤	11	6	54.5（23.4～83.3）	10	6	60.0（26.2～87.8）
口腔	12	5	41.7（15.2～72.3）	11	4	36.4（10.9～69.2）
眼睛	13	9	69.2（38.6～90.9）	12	8	66.7（34.9～90.1）
食管	11	4	36.4（10.9～69.2）	10	4	40.0（12.2～73.8）
胃肠道	11	9	81.8（48.2～97.7）	10	8	80.0（44.4～97.5）
肝脏	2	1	50.0（1.3～98.7）	1	1	100.0（2.5～100.0）
肺	12	5	41.7（15.2～72.3）	11	4	36.4（10.9～69.2）
关节筋膜	9	1	11.1（0.3～48.2）	8	1	12.5（0.3～52.7）

**表4:** 贝舒地尔治疗6个月时总体和既往芦可替尼治疗患者受累器官缓解情况

器官	总体（8例）			既往芦可替尼治疗（7例）		
	受累例数	缓解例数	缓解率［％（95％*CI*）］	受累例数	缓解例数	缓解率［％（95％*CI*）］
皮肤	6	5	83.3（35.9～99.6）	5	4	80.0（28.4～99.5）
口腔	7	6	85.7（42.1～99.6）	6	5	83.3（35.9～99.6）
眼睛	8	8	100.0（63.1～100.0）	7	7	100.0（59.0～100.0）
食管	6	6	100.0（54.1～100.0）	5	5	100.0（47.8～100.0）
胃肠道	6	6	100.0（54.1～100.0）	5	5	100.0（47.8～100.0）
肝脏	2	2	100.0（15.8～100.0）	1	1	100.0（2.5～100.0）
肺	8	5	62.5（24.5～91.5）	7	4	57.1（18.4～90.1）
关节筋膜	5	2	40.0（5.3～85.3）	4	2	50.0（6.8～93.2）

三、安全性

在治疗期间，15例（75.0％）报告了不良事件。最常见的不良事件包括肝酶升高（4例次，20.0％）、流行性感冒（4例次，20.0％）、新冠肺炎（3例次，15.0％）、肺部感染（3例次，15.0％）、腹泻（2例次，10.0％）。大多数不良事件为1级或2级，2例（10.0％）报告了3级及以上不良事件，分别为肺部感染和流行性感冒。1例患者在治疗期间因肺部感染死亡，该患者既往存在肺部感染，用药后症状加重。1例患者因原发疾病复发终止用药，随访期间内未发生贝舒地尔减量。2例既往巨细胞病毒和EB病毒感染患者，1例贝舒地尔治疗后出现EB病毒和巨细胞病毒再激活，未见新发EB病毒和巨细胞病毒感染。

四、青少年患者

本研究中，3例青少年患者均为12岁（男2例，女1例）。其中2例已经接受了超过6线治疗，1例接受过4线治疗。接受贝舒地尔治疗后，均获得部分缓解。1例患者累及8个器官，接受贝舒地尔治疗后，全部获得缓解。其余2例累及7个器官（皮肤、口腔、眼睛、食管、胃肠道、肺、关节筋膜），接受贝舒地尔治疗后，其中1例全部受累器官获得缓解，另1例皮肤、口腔、眼睛、关节筋膜获得缓解。LSS评分也均有临床意义的改善。在治疗期间，没有患者需要开始新的cGVHD系统治疗，也未出现肿瘤复发或死亡。所有不良事件均为1级或2级。

## 讨论

本研究首次在中国真实世界人群中对贝舒地尔治疗cGVHD的疗效和安全性进行了评估。研究中，所有患者均患有重度cGVHD，其中90.0％至少有4个器官受累，半数在启动贝舒地尔治疗前经历了至少4线治疗，为接受了多线治疗且累及多个脏器的重度cGVHD患者群体。结果表明，在该人群中，贝舒地尔具有良好的疗效和安全性。

既往研究已经证明，贝舒地尔对cGVHD具有良好的疗效。其中，ROCKstar研究是一项在美国开展的Ⅱ期研究，132例患者接受贝舒地尔200 mg每日1次或200 mg每日2次联合其他系统性治疗，中位随访14个月；中位年龄为56岁，67％患有重度cGVHD，52％至少4个器官受累，中位既往治疗线数为3线；两剂量组的ORR分别为74％和77％，LSS评分有临床意义改善的比例分别为59％和62％[15]。在中国开展的另一项Ⅱ期研究中，30例患者接受贝舒地尔200 mg每日1次治疗，中位随访12.9个月，ORR为73.3％，50.0％的患者实现了LSS评分有临床意义的改善[16]。与上述两项研究相比，本研究纳入的重度cGVHD患者比例更高、受累器官数更多、既往治疗线数更多（所有患者均为重度cGVHD，中位年龄34.5岁，90.0％的患者至少有4个器官受累，95％的患者肺部受累，中位既往治疗线数为4线，且75％的患者既往接受过芦可替尼治疗）。尽管患者病情更为复杂和难治，在既往免疫抑制剂或其他系统性治疗无效的情况下，联合贝舒地尔治疗仍获得ORR 90.0％，且总体不良反应发生率并未明显升高。这表明在真实世界中，贝舒地尔在既往接受多线治疗的重度cGVHD患者群体中，尤其是对芦可替尼治疗反应不佳的患者，具有良好的安全性及疗效。

本研究中没有患者达到完全缓解，这可能与多方面因素有关。首先，本研究中贝舒地尔的疗程和观察时间相对较短。此外，患者cGVHD病情严重，诊断cGVHD至开始贝舒地尔治疗的时间较长。部分患者在接受贝舒地尔治疗时，组织器官如肺和关节筋膜等已呈重度受累。严重的纤维化病变可能导致短期内难以获得良好的治疗反应[19]，因此需要更长期的治疗干预以解决难以逆转的纤维化问题。值得关注的是，本研究中各受累器官均有患者达到CR，包括肺部缓解率达到47.4％（CR率10.5％，PR率36.8％），这一比例高于先前国外2项Ⅱ期研究[20]合并分析中报告的ORR 37％（CR率14％，PR率23％）。肺部受累的19例患者中，13例既往接受过芦可替尼治疗，在治疗无缓解的情况下联合贝舒地尔治疗，高缓解率提示芦可替尼和贝舒地尔两种作用机制不同的药物联合使用可能具有协同效应[21]。本研究中肺部CR率仅10％，可能与启动贝舒地尔治疗时肺部严重程度评分高有关（1分5例，2分7例，3分7例）。研究表明，肺部评分越低，患者获得部分缓解或完全缓解的可能性越高[20]。有意思的是，在本研究中的2例启动治疗时肺部评分为1分患者均获得完全缓解，因此提示在疾病早期使用贝舒地尔可能带来更大获益。

贝舒地尔治疗对患者自评结局的改善作用也在本研究中得到印证。本研究显示，80.0％的患者在治疗3个月时表现出了具有临床意义的LSS评分改善，表明在真实世界中贝舒地尔治疗对改善患者自评结局的效应。在18例应答者中，77.8％的患者获得临床意义的改善，而2例非应答者也都获得了临床意义的改善。结果表明，无论是否应答，患者都获得了症状和社会功能的改善，提升了生活质量。

在本研究中，42.9％的患者获得糖皮质激素减量，启动贝舒地尔治疗到糖皮质激素减量的中位天数为30（10～60）d。在中位随访5.0个月期间内，尚无患者完全停用糖皮质激素。本研究中糖皮质激素减量患者占比低于既往两项Ⅱ期研究（56.7％、65％）[15]–[16]，可能与本研究纳入的患者人群特征以及随访时间有关。本组病例既往治疗线数较多，且cGVHD病情更为严重，且几乎所有患者都累及了肺部这样难以管理的器官，病情控制通常更加困难，对糖皮质激素依赖性更强，因此可能需要更长时间的治疗才能实现糖皮质激素减量和停用。此外，本研究的随访时间较短，因而部分患者尚未达到糖皮质激素减停。本研究中糖皮质激素剂量平均降低61.11％，高于ROCKstar研究中报告的45％[15]。

为进一步探索影响糖皮质激素减量的潜在因素，我们对14例联合使用糖皮质激素的患者进行了分析，基于接受贝舒地尔治疗后是否减低糖皮质激素剂量，将患者分为减量组（6例）和未减量组（8例）进行对比。受样本量所限，组间特征未显示出显著的统计学差异。然而值得注意的是，减量组与未减量组在某些特征上存在较大的数值差异。具体而言，减量组中急性移植物抗宿主病（aGVHD）病史的比例较低（16.7％对62.5％），而既往接受麦考酚酯和巴利昔单抗治疗的比例则较高（分别为66.7％对25.5％、50.0％对12.5％）。此外，其他基线疾病情况、器官受累情况、既往治疗以及在贝舒地尔治疗期间的合并治疗情况在两组之间均无明显差异。既往研究结果支持aGVHD病史与更差的cGVHD预后相关，因此患者的治疗可能更为复杂和困难[22]。而既往治疗方案与糖皮质激素减量的关系，仍需进一步研究探讨。

对于既往接受过芦可替尼治疗的患者，本研究进一步证实了贝舒地尔的疗效。在这15例患者中，ORR为86.7％，有73.3％的患者在3个月时获得了临床意义的改善。本研究中的治疗与以往临床试验停用芦可替尼洗脱不同，患者均为芦可替尼与贝舒地尔联合用药，随着病情稳定，芦可替尼剂量逐渐减低。既往已有多项真实世界研究证实贝舒地尔与芦可替尼联合使用在治疗难治性cGVHD时有着较好的耐受性、安全性和疗效[23]–[25]。对芦可替尼反应不佳的患者，联合使用贝舒地尔可能是一个可行的治疗选择，在达到缓解后芦可替尼可逐渐减停[23]。本研究进一步证实，在芦可替尼治疗失败的患者中加用贝舒地尔仍然能获得良好的临床获益。cGVHD复杂的病理生理机制涉及多种炎症和纤维化通路，常需多药联合治疗。联合使用贝舒地尔和芦可替尼的理论基础在于其作用机制互补，通过靶向不同通路可能产生协同效应。芦可替尼作为一种JAK1/2抑制剂，主要抑制细胞因子介导的免疫过激反应；而贝舒地尔作为一种ROCK2抑制剂，则具有免疫调节和抗纤维化的双重作用[11]–[12]。与单药治疗相比，双重抑制这些通路可能提高疗效，同时，可能降低通路间代偿性激活的风险，延缓耐药的发生，为cGVHD患者带来更持久的临床获益和更广阔的治疗前景[27]。

安全性方面，贝舒地尔在真实世界显示了较好的耐受性，大多数不良事件为轻度至中度。仅1例患者因原发疾病复发终止用药。研究中未观察到新的不良事件，血液学毒性及病毒再激活风险较低，安全性结果与既往临床研究[15]–[16]一致。

截至2025年1月13日，本研究中15例患者仍在接受贝舒地尔治疗，未出现新的死亡事件或原发疾病复发事件。1例患者因个人意愿停止治疗，1例患者失访。12例患者（80.0％）的LSS评分显示出临床意义的改善，平均降低17.60分（标准差为14.06）。本研究中获得PR的14例患者中有13例在2025年1月13日仍维持PR，1例出现疾病进展（PD），1例在本研究中评估为疾病稳定（SD）的患者在2025年1月13日前获得了PR。除1例肺部受累的患者出现疾病进展外，本研究中皮肤、口腔、眼睛、胃肠道、肺部、肝脏以及关节筋膜受累后达到PR/CR的患者在长期随访中仍然维持缓解。部分患者随着治疗的持续实现缓解，分别有2例皮肤和眼睛SD的患者转为PR，3例口腔SD患者转为CR，2例胃肠道SD患者转为CR。

本研究存在以下局限性：①样本量较小，在一定程度上限制了结果的普遍性和可推广性。②随访时间较短，无法全面反映贝舒地尔在长期治疗中的效果及安全性。cGVHD作为一种慢性病，其治疗效果的评估需要较长时间的观察，以便更好地理解治疗对患者生活质量及长期预后的影响。因此，未来应考虑扩大样本量并延长随访时间，以期更全面地评估该药物的长期疗效与安全性。
